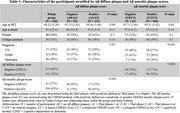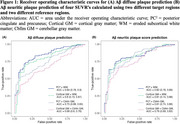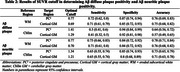# Amyloid quantification in the oldest‐old: selecting regions for optimizing correspondence between pathology at autopsy and amyloid PET

**DOI:** 10.1002/alz70855_106161

**Published:** 2025-12-24

**Authors:** Jiaxin Yu, Davis C. Woodworth, Evan Fletcher, Dana E. Greenia, Syed A. Bukhari, Thomas J. Montine, María M. M. Corrada, Claudia H. Kawas, Charles Decarli, Tianchen Qian, Seyed Ahmad Sajjadi

**Affiliations:** ^1^ University of California, Irvine, Irvine, CA, USA; ^2^ University of California, Davis, Davis, CA, USA; ^3^ Stanford University, Stanford, CA, USA; ^4^ Department of Pathology, Stanford University School of Medicine, Stanford, CA, USA; ^5^ University of California Irvine, Irvine, CA, USA; ^6^ Department of Neurology & Imaging of Dementia and Aging Laboratory, University of California Davis, Sacramento, CA, USA

## Abstract

**Background:**

Positron emission tomography (PET) is the current gold standard for assessing amyloid burden *in vivo* and is often quantified using standardized uptake value ratios (SUVR). However, there are multiple ways to compute SUVRs, including using different regions of interest (ROI) or different reference regions. Choice of ROI and reference regions becomes especially important in the oldest‐old, where advanced atrophy and contraindications for MRI can complicate amyloid quantification for certain regions. This study compared the performance of four different SUVR calculation methods against the gold standard of postmortem pathology.

**Method:**

We used data from participants from *The 90+ Study* who had both florbetapir PET and postmortem neuropathological assessments. PET scans were re‐aligned to an older age template using a custom MRI‐free pipeline. We examined two neuropathological amyloid outcomes: presence of diffuse plaques, defined as Thal phase 3 or higher, and neuritic plaque positivity, defined as a moderate or greater CERAD neuritic plaque score. Four SUVRs were calculated by combining two different ROIs—combined posterior cingulate and precuneus (PC²) and cortical gray matter (CorticalGM)—with two different reference regions—white matter (WM) and cerebellar gray matter (CbllmGM). We assessed the predictive performance of each SUVR using ROC analysis and applied Youden's index to determine optimal cutoffs.

**Result:**

Table 1 displays participant characteristics (*N* = 165). Average age at PET was 94 years and average age at death was 97 years. SUVRs using WM as the reference region consistently outperformed those using CbllmGM in predicting diffuse and neuritic plaque positivity (Figure 1). PC²+WM showed the highest area under the curve (AUC) of 0.84 (95% CI: 0.78–0.90) for diffuse plaques and 0.82 (95% CI: 0.76–0.89) for neuritic plaque positivity. The optimal cutoff for PC²+WM was determined to be 0.77, with a sensitivity of 0.72 (95% CI: 0.62–0.80) and a specificity of 0.87 (95% CI: 0.74–0.94) for predicting the presence of diffuse plaque (Figure 2). These findings remained robust in sensitivity analyses.

**Conclusion:**

PC²+WM was the best combination of regions among the four SUVR methods for predicting amyloid diffuse and neuritic plaques in this group of oldest‐old participants.